# ProbeMaker: an extensible framework for design of sets of oligonucleotide probes

**DOI:** 10.1186/1471-2105-6-229

**Published:** 2005-09-19

**Authors:** Johan Stenberg, Mats Nilsson, Ulf Landegren

**Affiliations:** 1 Department of Genetics and Pathology, Rudbeck Laboratory, Uppsala University, Se-751 85, Uppsala, Sweden

## Abstract

**Background:**

Procedures for genetic analyses based on oligonucleotide probes are powerful tools that can allow highly parallel investigations of genetic material. Such procedures require the design of large sets of probes using application-specific design constraints.

**Results:**

ProbeMaker is a software framework for computer-assisted design and analysis of sets of oligonucleotide probe sequences. The tool assists in the design of probes for sets of target sequences, incorporating sequence motifs for purposes such as amplification, visualization, or identification. An extension system allows the framework to be equipped with application-specific components for evaluation of probe sequences, and provides the possibility to include support for importing sequence data from a variety of file formats.

**Conclusion:**

ProbeMaker is a suitable tool for many different oligonucleotide design and analysis tasks, including the design of probe sets for various types of parallel genetic analyses, experimental validation of design parameters, and *in silico *testing of probe sequence evaluation algorithms.

## Background

Increasing numbers of methods are being developed for parallel nucleic acid analyses for different purposes. Many of these methods employ sets of oligonucleotide probes or probe pairs that hybridize to the sequences targeted for analysis, allowing the probe sequences to be acted upon by one or more enzymes, creating new molecular species that reflect the presence or nature of the different target sequences. The reaction products generally contain identifying sequences or other features that allow the separation of signals originating from different targets. This is the case in methods such as the multiplex oligonucleotide ligation assay (OLA) [1], the multiplex ligation-dependent probe amplification assay (MLPA) [2], the RNA- and cDNA-mediated annealing, selection, extension and ligation assays (RASL, DASL) [3,4], the GoldenGate genotyping assay [5], multiplex minisequencing [6], and the padlock or molecular inversion probe assay [7,8]. The latter method has been used to genotype more than 10,000 single nucleotide polymorphisms (SNPs) in multiplex. Another method that utilizes sets of oligonucleotide probes for multiplex processing of nucleic acid molecules is the selector amplification technique. This technique uses partially double-stranded oligonucleotides, called selectors, to circularize a selection of restriction fragments from total genomic DNA, and it incorporates a general sequence motif that allows parallel amplification of all circularized fragments using a single primer pair [9].

With molecular solutions to many tasks of highly parallel genetic analysis now at hand, other factors become limiting, such as the design and the synthesis of reagents. In the work presented here, we address the problem of large-scale probe design. When large numbers of probes are combined, the risk for unintended interactions between probes and targets must be considered. This risk places strict requirements on the design of sets of probes to be used together. In particular, it is important that probes do not contain sequences that result in the production of detectable signal from any probe in the absence of its cognate target molecule, or that otherwise interfere with the activity of other probes in the set. Due to these and other constraints and the many possible alternative probe sequences to evaluate, the difficulty of designing probe sets increases rapidly with the size of the probe sets.

Many computer programs exist for the design of oligonucleotide probes such as PCR primers [10-12], microarray probes [13,14], and more [15]. These programs define algorithms to evaluate the risk of primer or probe sequences being involved in undesired interactions such as probe homo- or heterodimer formation, cross-hybridization, false priming, *etc*. However, the available programs are generally limited in scope, and are not applicable to the task of designing sets of complex probes containing multiple sequence elements.

The ProbeMaker software presented herein is a framework for computer-assisted design and analysis of sets of oligonucleotide probe sequences composed of several functional sequence elements. As the composition of probes and the constraints imposed on sets of probes vary between applications, this framework has been constructed to support the design of different types of probes using application-specific constraints, as defined by the user. ProbeMaker takes as input a set of target sequences and a number of sets of so-called 'tag' sequences. These tag sequences may serve as targets for restriction digestion, as binding sites for amplification primers or fluorescent detection probes, or as identification codes for individual amplification products that are decoded by hybridization to oligonucleotide arrays [16]. Probes are designed for each target by construction of target-specific sequences and addition of tag sequences according to rules specified by the user. Different combinations of sequence elements are evaluated for each probe, and a set of probe sequences is created that satisfies user-defined criteria.

## Implementation

The main objectives in the development of ProbeMaker were to provide a framework that is flexible, in the sense that it should support design of oligonucleotide probes for different purposes, and extensible, in that it should be possible to add support for designing new types of probes and to add new types of design constraints. Furthermore, the software should be adaptable to new applications, and it should have the potential to import sequence data from a variety of sources.

The flexibility is provided by the target and probe sequence data structures used. Each target defines two template sequences that are used to construct target-specific sequences (TSSs) to use in the corresponding probe. Each probe is made up of two such TSSs and a number of tag sequences, which may be located 5' of, between, or 3' of the TSSs. As TSSs may be of zero length, this system allows the design of many different types of probes. Support for more than two TSSs per probe was not deemed necessary as this is not used in any current methods. Furthermore, targets may be grouped, allowing the program to perform selection of tag sequences based on the relations of target sequences, for example variants of the same polymorphic sequence.

The extensibility is realized by using an extension mechanism for much of the functionality. Extensions are constructed in the form of Java classes that implement defined interfaces and may be loaded into the framework at run-time. This mechanism allows the addition of new target types and support for different formats for sequence input and output, as well as design constraints and acceptor schemes, the function of which will be described below.

ProbeMaker may be run through a graphical user interface or from the command line. For the graphical user interface, a set of target sequences and sets of tag sequences are provided as input by the user. Application-specific parameters for probe design and evaluation are set through the user interface. When running ProbeMaker from the command line, a project file defining all sequences and parameters is used as input.

The potential for supporting different file formats is provided by using the sequence input system of the MolTools Java library [17]. A combination of components for sequence file parsing, sequence notation conversion, and post-import modifications are used to allow creation of sets of any type of target from a variety of sequence file formats, with the possibility to carry out other operations on the imported data, such as selecting which position within the target sequence to design probes for, or to group or sort sequences based on some particular property.

## Results

**Figure 1:**
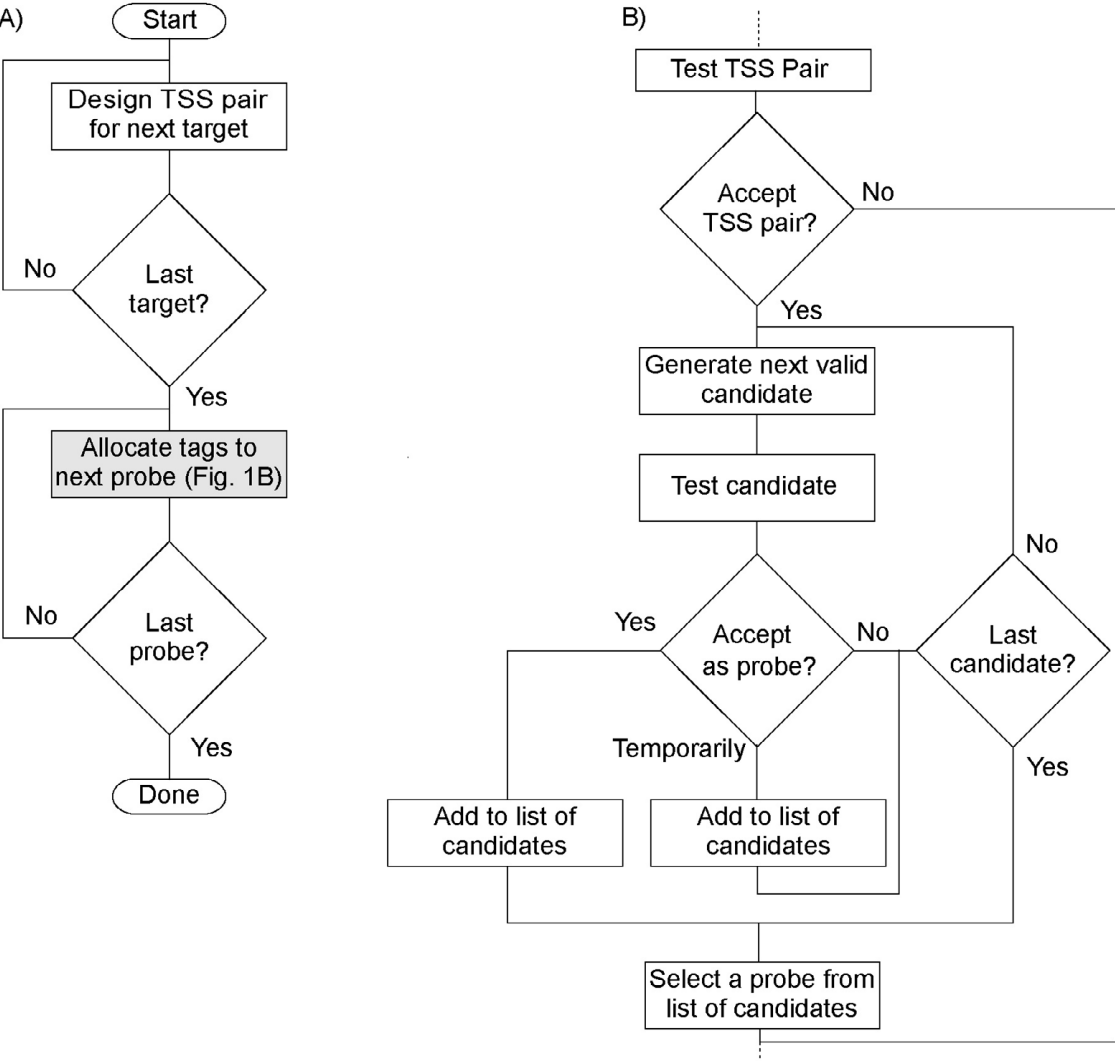
**Schematic description of the probe set design procedure**. A) Target-specific sequences are first designed for all targets. Tags are then added to each TSS pair in sequence to form complete probes. B) Prior to tag allocation, each TSS pair is evaluated using selected constraints and the current acceptor scheme. Accepted TSS pairs are used to create a series of probe candidates using each valid combination of tags in turn. This procedure is stopped if an acceptable candidate is found, or when all candidates have been tested. A probe is then selected from the list of accepted or temporarily accepted candidates, using the current selector scheme.

For a given set of targets, and a number of sets of tag sequences, ProbeMaker performs two tasks (Figure 1A). Firstly, TSSs are constructed for each target as determined by the target type in use, forming the basis for a probe for that target. Secondly, tag sequences are added to each probe sequentially in a pattern specified by the user. During this procedure, different combinations of tags are evaluated for each probe in order to find one that satisfies specified design constraints.

### Target-specific sequence construction

The TSSs of each probe are constructed to be complementary to the template sequences defined by the target, with sequence length chosen within a specified length interval to yield a melting temperature (T_m_) as close as possible to a specified preferred T_m _value. The T_m _calculations are performed using a nearest neighbor model [18-20]. The model implementation used here does not take into account the influence of dangling ends or possible stacking interactions between the two probe ends, as these are not always known at this stage of the design. It is possible to use target types that strictly determine the TSS length, which is useful *e.g. *if other software has been used to find suitable sequences for probe-target hybridization.

### Tag selection

After TSS construction, each probe is designed in turn by generation, evaluation, and selection of probe candidates as follows (Figure 1B).

1) The target-specific sequences of the probe are evaluated according to selected design constraints. If found acceptable the process proceeds, otherwise the probe is skipped and reported as a failure.

2) A probe candidate is generated by allocating one tag from each set of tag sequences. This candidate is evaluated according to selected design constraints and ranked on a three-level scale. Based on this rank, the candidate is either accepted, stored in a temporary list, or rejected. This step is iterated, generating a new candidate each time, until all possible tag combinations have been tried or until a candidate has been accepted.

3) One probe is selected from the list of temporarily accepted candidates and any finally accepted candidate.

Probe candidates are constructed by the selection of tags from the provided tag sets based on the selection mode of each tag set. There are five selection modes available.

• A unique tag for every probe

• A common tag for all probes

• A tag common for probes within a group, but unique among groups

• A different tag for each probe in a group, same set of tags used for all groups

• Any tag, regardless of use in other probes

Optionally, a spacer tag may be included to extend any probe that is shorter than a specified length, if probes of identical lengths are desired. Several possible tag combinations may exist for each probe, depending on the selection mode and what tags have been used previously in the probe set. Also, during candidate testing, certain tags may be found unsuitable for use in a particular probe and be excluded from the selection procedure in order to reduce the number of candidates that need to be tested for that probe.

For testing and evaluation of target-specific sequences and probe candidates, the user selects tests that are suitable for the type of probes currently being designed. These tests are incorporated into the framework as extensions. Typically, tests will check for potential unwanted base-pairing interactions within a probe, between a probe and its target, between probes, or between a probe and the targets of other probes. Each test may generate warnings or errors for a candidate; these are then used to rank that candidate. Candidates are by default of the highest rank, warnings reduce this to the intermediate level while errors result in the lowest rank.

Criteria for accepting probe candidates and for choosing among stored candidates are specified by the user by selecting an acceptor scheme and a selector scheme. The acceptor schemes provided with the program include one that will temporarily store all candidates of intermediate or highest rank, and one that will accept candidates of the highest rank, while temporarily storing those of intermediate rank. When probes are designed in groups of two, an exhaustive tag selection mode is available. In this mode, the first probe of a group is not finally determined until an acceptable candidate has been found for the second probe. Both probes are skipped if acceptable candidates cannot be found.

### Limitations

Probe design is limited by the amount of available memory, and the amount of time required. Using 500 MB of RAM, it is possible to design probes for at least 20,000 targets. However, when using tests for inter-probe interactions the design time grows exponentially with the number of targets and quickly becomes more limiting than memory. The time required for ProbeMaker to complete a design job is influenced by many factors and is difficult to model and predict. Briefly, the design time depends on the total number of candidates that are generated and the time required for the selected tests to be performed on each generated candidate. The maximum number of candidates generated depends on the size and selection mode of the tag sets used in the design, while the time required for testing of each candidate depends on the tests that are performed and the number of targets/probes being designed.

To illustrate actual time requirements, we set up a design of allele-specific pairs of padlock probes for 1000 random SNP target sequences, allocating to each probe one common primer, either of two allele-specific primers, and one target-specific hybridization tag from a set of 1000 random 20-mers. Without constraints, this design required 8 seconds to complete on a desktop computer system (Intel Pentium 4, 2.5 GHz). When testing for the risk of false ligation using an adaptation of the false-priming algorithm described by Kaderali *et al*. [15], the same design required 10.5 minutes to complete.

## Discussion

A number of recently developed methods for nucleic acid analyses allow large sets of oligonucleotide probes to be used in parallel for simultaneous interrogation of many qualities of a sample. These methods require design of large numbers of oligonucleotide probes. Computer programs commonly used to design various types of oligonucleotides [10-15] define a repertoire of criteria, and algorithms to evaluate oligonucleotides based on these criteria. However, the available programs are mainly dedicated for the design of amplification or sequencing primers or microarray probes, and most programs can not readily be modified for new uses.

In this work, we present a framework for computer-assisted design and analysis of sets of oligonucleotide probes. The ProbeMaker software allows the design of sets of any type of probes with up to two elements that are complementary to the target sequence and that include a number of other sequence elements. Furthermore, ProbeMaker is equipped with an extension mechanism that allows the incorporation of new design criteria as well as criteria described in previous works. Similarly, it is possible to define new types of targets, which will allow the design of new types of probes, including probes for non-nucleic acid targets, such as pairs of oligonucleotides to be attached to antibodies or other affinity reagents for protein analyses by proximity ligation [21,22].

## Conclusion

ProbeMaker enables constraint-based design of large sets of probes. Besides facilitating the deployment of large-scale assays, this can be used to systematically vary design criteria in order to experimentally optimize design parameters. Furthermore, the flexibility and extensibility of this framework makes it suitable for *in silico *comparison and evaluation of different oligonucleotide analysis algorithms, and it could act as a common platform for further development within the field.

## Availability and requirements

**Project name: **ProbeMaker

**Project home page: **

**Operating system(s): **Platform independent

**Programming language: **Java

**Other requirements: **Java 1.4 or higher, MolTools and AppTools libraries (provided with ProbeMaker and available under the GNU LGPL License from )

**License: **GNU GPL

**Any restrictions to use by non-academics: **No

## Authors' contributions

JS designed and implemented the software and drafted the manuscript. MN and UL conceived of and supervised the work. All authors read and approved the final manuscript.
